# Identifying Synergistic Mechanisms of Multiple Ingredients in Shuangbai Tablets against Proteinuria by Virtual Screening and a Network Pharmacology Approach

**DOI:** 10.1155/2020/1027271

**Published:** 2020-01-06

**Authors:** Xiaoqin Ma, Meixiang Yu, Chenxia Hao, Wanhua Yang

**Affiliations:** ^1^Department of Pharmacy, Ruijin Hospital, Shanghai Jiaotong University School of Medicine, Shanghai 200025, China; ^2^Department of Pharmacy, Ruijin Hospital North Affiliated to the Shanghai Jiao Tong University Medical School, Shanghai 201801, China

## Abstract

Shuangbai Tablets (SBT), a traditional herbal mixture, has shown substantial clinical efficacy. However, a systematic mechanism of its active ingredients and pharmacological mechanisms of action against proteinuria continues being lacking. A network pharmacology approach was effectual in discovering the relationship of multiple ingredients and targets of the herbal mixture. This study aimed to identify key targets, major active ingredients, and pathways of SBT against proteinuria by network pharmacology approach combined with thin layer chromatography (TLC). Human phenotype (HP) disease analysis, gene ontology (GO) analysis, Kyoto Encyclopedia of Genes and Genomes (KEGG) pathway enrichment analysis, and molecular docking were used in this study. To this end, a total of 48 candidate targets of 118 active ingredients of SBT were identified. Network analysis showed PTGS2, ESR1, and NOS2 to be the three key targets, and beta-sitosterol, quercetin, and berberine were the three major active ingredients; among them one of the major active ingredients, quercetin, was discriminated by TLC. These results of the functional enrichment analysis indicated that the most relevant disease including these 48 candidate proteins is proteinuria, SBT treated proteinuria by sympathetically regulating multiple biological pathways, such as the HIF-1, RAS, AGE-RAGE, and VEGF signaling pathways. Additionally, molecular docking validation suggested that major active ingredients of SBT were capable of binding to HIF-1A and VEGFA of the main pathways. Consequently, key targets, major active ingredients, and pathways based on data analysis of SBT against proteinuria were systematically identified confirming its utility and providing a new drug against proteinuria.

## 1. Introduction

Proteinuria is an established marker of kidney damage and a risk factor for progression of chronic kidney disease (CKD) [1–3]. Albuminuria is classified as an indicator of advanced CKD [4] according to the Kidney Disease Improving Global Outcome (KDIGO) clinical practice guideline for glomerulonephritis [5]. Macroalbuminuria (>300 mg/24 h) is an extremely common feature in diabetic patients with advanced CKD, with a frequency range between 33% in stage 3B and nearly 100% in stage 5 [4, 6]. Proteinuria is associated with considerable mortality not only [7, 8] in the diabetic and nondiabetic population but also with cardiovascular events [9]. Therefore, controlling urinary albumin levels is important in treating proteinuria and its associated primary diseases [10]. Indeed, proteinuria pathogenesis is complex and multifactorial due to its relation with various diseases. However, some studies have found that proteinuria is associated with podocyte injury, endothelial cell injury, and an imbalance in the regulation of vascular endothelial growth factor (VEGF) [11–14].

Despite some advancements in the understanding of proteinuria pathogenesis, there is still a lack of therapies that work against proteinuria other than using the renin-angiotensin system (RAS) inhibitor as the gold standard [5], such as angiotensin-converting enzyme inhibitors (ACEI) or angiotensin receptor blockers (ARB). Unfortunately, RAS blocking slows down but does not preclude the progression of kidney disease [15] and increases the potential risk of hyperkalemia [16]. Immunosuppressive treatment with corticosteroids has shown some effect in remission of proteinuria although a certain percentage of patients do not respond to treatment and are labeled steroid resistant [17]. Glucocorticoid therapy can be resistant or dependent and can also cause many adverse reactions [17, 18]. Thus, it is crucial to search for more safe and effective drugs in treating proteinuria and its primary diseases based on their pharmacological mechanisms [15].

Recently, Traditional Chinese Medicine (TCM) has played an important role in the treatment of proteinuria via its multicomponent, multitarget, and multipathway approach [15, 19–23]. Shuangbai Tablet (SBT) is a classic TCM, which was approved by the Shanghai Food and Drug Administration. It has achieved satisfactory results for the treatment of mild to moderate proteinuria and its primary disease for more than twenty years in Ruijin Hospital, Shanghai Jiao Tong University [24]. SBT is composed of 15 traditional Chinese herbs: *Imperatae Rhizoma* (IRH), *Hedysarum multijugum Maxim* (HMM), *Acanthopanax senticosus* (ASE), *Caragana sinica Rehd* (CSR), *Achyranthis Bidentatae Radix* (ABR), *Radix Paeoniae Rubra* (RPR), *Paeoniae Radix Alba* (PRA), *Fructus lipuidambaris* (FRU), *Lycopi Herba* (LHE), *Tetrapanacis Medulla* (TME), *Polygonum perfoliatum L* (PPL), *Sambucus williamsii Hance* (SWH), *Dipsaci Radix* (DRA), *Stephaniae Tetrandrae Radix* (STR), and *Chuanxiong Rhizoma* (CRH). Furthermore, some single-flavor herbs or ingredients in SBT, such as Astragaloside IV of HMM, paeoniflorin of PRA, and CRH have also been revealed an exact therapeutic effect against proteinuria [25–27].

**Figure 1:**
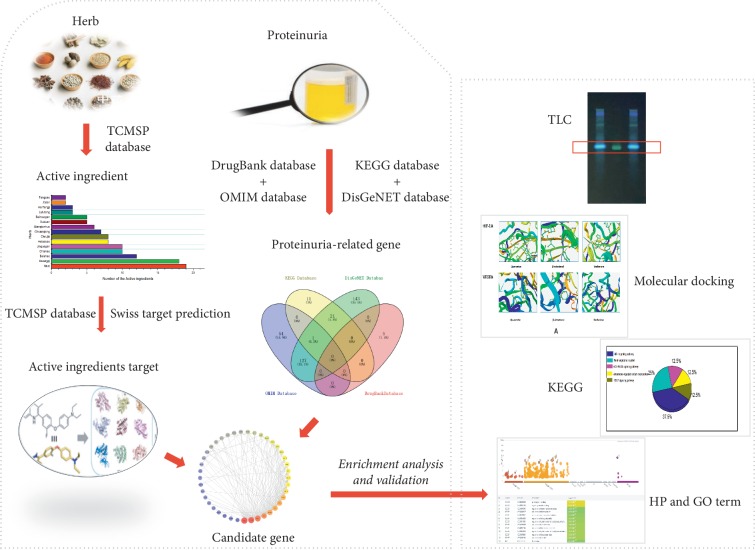
Flow diagram of this study. Left: summary of the identification of representative ingredients of SBT and genes with efficacy against proteinuria. Right: summary of the determination and validation of the pharmacological mechanisms of SBT.

However, active components and potential pharmacological mechanisms of action of SBT against proteinuria have not been identified. In this study, human phenotype (HP) disease analysis, gene ontology (GO) analysis, Kyoto Encyclopedia of Genes and Genomes (KEGG) pathway enrichment analysis, and molecular docking were applied to investigate the bioactive constituents and the underlying mechanisms of SBT for the treatment of proteinuria, combined with thin layer chromatography (TLC), to identify a single active ingredient. The flow diagram of this study is shown in Figure 1.

## 2. Materials and Method

SBT (MUST-1901001) was provided by Ruijin Hospital, Shanghai Jiaotong University, China. Quercetin (MUST-100081-200406, purity ≥97.3%) was purchased by China National Institute for Biological Products Standard. Reagent methanol (MUST-20180105), hydrochloric acid (MUST-20190104), ethyl acetate (MUST-20170609), formic acid (MUST-20140212), and toluene (MUST-20160106) supplied by Lingfeng Chemical Reagent Co., Ltd. (Shanghai, China). Ethanol (MUST-20190530) and aluminum trichloride (MUST-20100127) were supplied by Klings and Qiangshun Chemical Reagent Co., Ltd. (both in Shanghai, China), respectively. Distilled water was used, and all other reagents were of analytical reagent.

### 2.1. Active Ingredients Database Building

The active ingredients of each herb in SBT were obtained using TCMSP (http://lsp.nwsuaf.edu.cn/tcmsp.php) [28], according to bioavailability (OB) ≥30% [20] and drug-likeness (DL) ≥0.18 [29], as well as TCMGeneDIT (http://tcm.lifescience.ntu.edu.tw/) [30] and related studies [31–37].

### 2.2. Targets of Active Ingredients Database Building

The targets of active ingredients were searched in TCMSP and SwissTargetPrediction (http://www.swisstargetprediction.ch) databases with *Homo sapiens* [38, 39]. SwissTargetPrediction is a molecular similarity match tool based on simplified molecules which contain a larger collection of 376,342 compounds known to be experimentally active on an extended set of 3068 macromolecular targets [38].

### 2.3. Genes for Building a Proteinuria Database

Genes associated with proteinuria were searched on four existing human disease resources, including Online Mendelian Inheritance in Man Database (OMIM, http://www.omim.org/) [40], the Kyoto Encyclopedia of Genes and Genomes Pathway Database (KEGG, https://www.kegg.jp/) [41], DisGeNET (https://www.disgenet.org/) [42] database, and DrugBank database (https://www.drugbank.ca/) [43]. After deletion of duplications, Uniprot ID of each target of proteinuria was queried by the Uniport database (https://www.uniprot.org/) [44].

### 2.4. Candidate Target Identification and “Herbs-Active Ingredients-Candidate Targets” Comprehensive Network Construction

Candidate targets against proteinuria were identified by matching the targets of the active ingredients of SBT and the genes related to proteinuria. Next, the “herbs-active ingredients-candidate targets” comprehensive network was established using Cytoscape software (v3.7.1; https://www.nigms.nih.gov/). We calculated the number of overlapped active ingredients in 15 herbs, as well as the number of overlapped candidate targets in active ingredients, and the top three were considered as the major active ingredients and the key targets, respectively.

### 2.5. Candidate Target Interacting Protein Network Construction

The interacting protein candidate targets against proteinuria were obtained from the String database (https://string-db.org/) [45–47] in which protein-protein interaction (PPI) information between two proteins or multiple proteins was given, and the combined score of each pair of interacting proteins was greater than or equal to 0.4 [47]. Next, the “candidate targets-proteins” network was built by Cytoscape 3.7.1 software [48–50]. Calculating the average degree of the nodes by using Network Analyzer plugin, all the nodes of the network represented central targets for enrichment analysis [50].

### 2.6. Enrichment Analysis Methods and Molecular Docking Simulation

#### 2.6.1. Enrichment Analysis Method

We performed HP and online GO term enrichment analysis by placing all central targets into g:Profiler (https://biit.cs.ut.ee/gprofiler/) [51–53]. KEGG pathway enrichment analysis was performed by placing those targets into ClueGo plugin (ClueGo v2.5.4 + ClueGoPedia v1.5.4) [54].

HP enrichment analysis is a web that provides a standardized vocabulary of phenotypic abnormalities encountered in human disease which can reveal the correlation between central targets and proteinuria. GO term is able to classify functions into three aspects: molecular functions (molecular activities of gene products), cellular components (where gene products are active), and biological processes (pathways and larger processes made up of the activities of multiple gene products).

#### 2.6.2. Molecular Docking

After identifying the pathways, in order to certify the reliability of the above enrichment analysis, we docked the factors in the pathways and the major active ingredients by SystemsDock (http://systemsdock.unit.oist.jp/) [55, 56], which classified the major active ingredients' activity by the criterion: a docking score greater than 5.52 or the score of the local ligands [55].

SystemsDock is a web server for network pharmacology-based prediction and analysis, which applies high-precision docking simulation and molecular pathway map to comprehensively characterize the ligand selectivity and to illustrate how a ligand acts on a complex molecular network. Structure files in the formats of 2D of the major active ingredients download from PubChem [57] (https://pubchem.ncbi.nlm.nih.gov/) and protein PDB ID of the factors in the pathways download from String and PDB database [58] (https://www.rcsb.org/).

### 2.7. Quercetin TLC Validation

#### 2.7.1. Sample of SBT

30 tablets of SBT were dissolved in 50 ml (methanol-hydrochloric acid (25%) (4 : 1)) solution and heated under reflux for 1 hour. The residue was dissolved (20 ml distilled water) and extracted twice (20 ml of ethyl acetate). Next, ethyl acetate solution was washed (10 ml of distilled water), discarding the aqueous solution. Finally, the dissolved residue (2 ml of methanol) was prepared as SBT sample.

#### 2.7.2. Standard Sample of Quercetin

Standard sample of quercetin (100081–200406, 2 mg/ml) was prepared by dissolving the solutes in methanol.

#### 2.7.3. Thin Layer Chromatography

According to the thin layer chromatography (General Rule 0502, Part IV, Chinese Pharmacopoeia) test, the solutions of SBT samples (10 *μ*l) and quercetin standard sample (2 *μ*l) were spotted manually on the chromatographic plates. The mixture of toluene + ethyl acetate + formic acid (25 : 20 : 1) was used as the mobile phase. The plates were developed vertically at room temperature (20°C) to a distance of 10 cm and then dried for 20 h at room temperature (20°C). Finally, it was sprayed with 10% aluminum trichloride in ethanol and placed under ultraviolet light (356 nm) for examination.

## 3. Results

### 3.1. Active Ingredients of SBT

**Figure 2:**
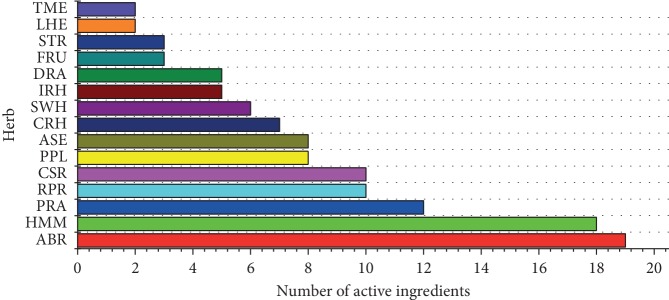
The number of active ingredients in SBT.

A total of 118 active ingredients (Supplementary [Supplementary-material supplementary-material-1]) were identified from 15 herbs in this study. The number of active ingredients in IRH, HMM, ASE, CSR, ABR, RPR, PRA, FRU, LHE, TME, PPL, SWH, DRA, STR, and CRH was 5, 18, 8, 10, 19, 10, 12, 3, 2, 2, 8, 6, 5, 3, and 7, respectively (shown in Figure 2). The top three herbs with higher active ingredients were ABR, HMM, and PRA (see Figure 2).

### 3.2. The 8981 Targets of Active Ingredients

**Figure 3:**
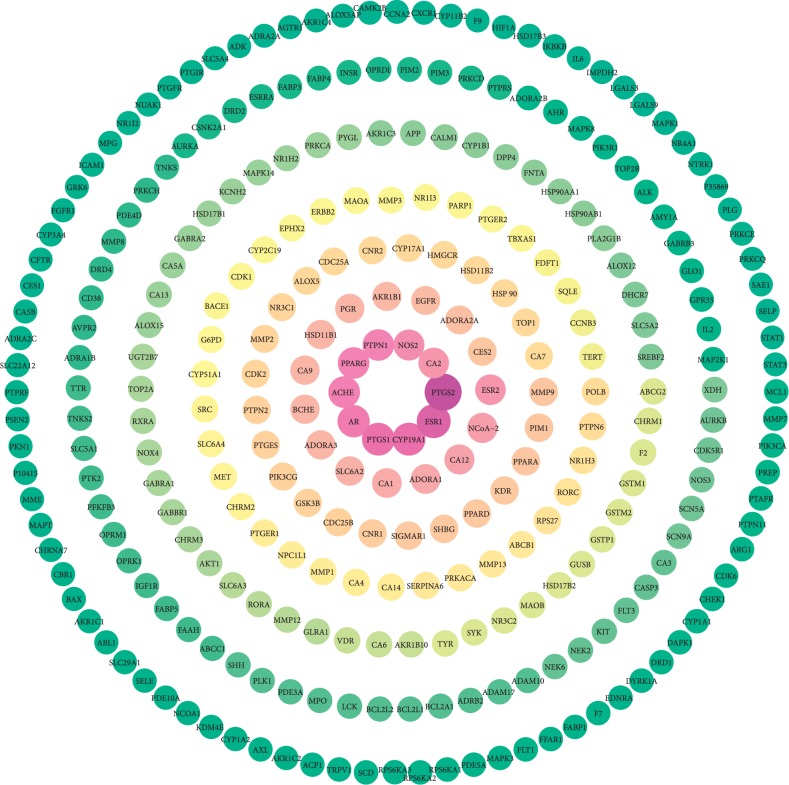
The targets with a frequency greater than 10; the color of the targets is shown in a gradient from red to green according to descending order of the frequency value with a yellow excess in the middle.

A total of 8981 human disease targets of the active ingredients in SBT (Supplementary [Supplementary-material supplementary-material-1]) were identified, among them 289 targets with the active ingredients frequency greater than 10 are shown in Figure 3. The top three active ingredients with higher number of targets are beta-sitosterol, quercetin, and berberine, and the top 10 overlapped targets are PTGS2, ERS1, CYP19A1, PTGS1, AR, ACHE, PPARG, PTPN1, NOS2, and CA2. The information of these targets is listed in Table 1.

### 3.3. Genes Associated with Proteinuria

A total of 523 human disease genes associated with proteinuria (Supplementary [Supplementary-material supplementary-material-1]) were identified from four databases. The number of genes in the OMIM, KEGG, DisGeNET, and DrugBank databases was 182, 33, 303, and 5, respectively.

### 3.4. Candidate Target and “Herbs-Active Ingredients-Candidate Targets” Network

**Figure 4:**
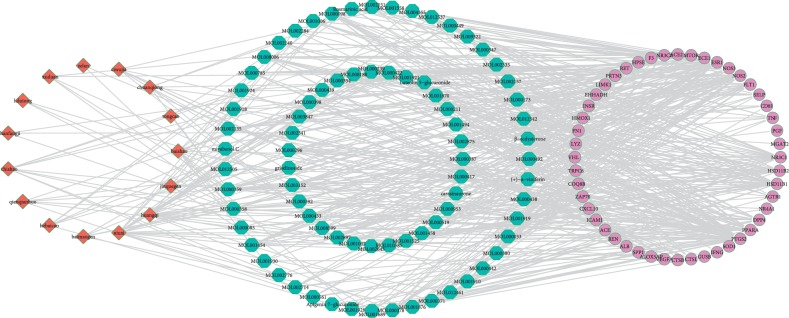
The “herbs-active ingredients-candidate targets” network; the red diamond nodes represent the herbs, the blue hexagon nodes represent the active components, and the purple circle nodes represent the targets.

A total of 48 candidate targets (Supplementary [Supplementary-material supplementary-material-1]) were shown to have pharmacological effects against proteinuria. The comprehensive network of “herbs-active ingredients-candidate targets” had 141 nodes and 524 edges, as determined by the Cytoscape 3.7.1 software (see Figure 4). There were 16 active ingredients with the number of targets exceeding 8, of which, the top three active ingredients (beta-sitosterol (MOL000358), quercetin (MOL000098), and berberine (MOL001454)) were represented as the three major active ingredients. Information of those 16 active ingredients, including their inclusion in herbs and the number of targets they regulate, is listed in Table 2. As shown in Table 2, beta-sitosterol is contained in 9 herbs, such as ABR, HMM, and PRA; quercetin is contained in HMM and PPL; berberine is contained in ABR.

There were 10 candidate targets with the number of active ingredients exceeding 14 (Table 3), of which the top 3 candidate targets (prostaglandin G/H synthase 2 (PTGS2), estrogen receptor (ESR1), and nitric oxide synthase (NOS2)) were represented as three key targets. As shown in Table 3, PTGS2, ESR1, and NOS2 are regulated by 74, 59, and 37 kinds of active ingredients, respectively, and all those three key targets can be regulated by three major active ingredients.

### 3.5. Candidate Targets-Proteins Network

**Figure 5:**
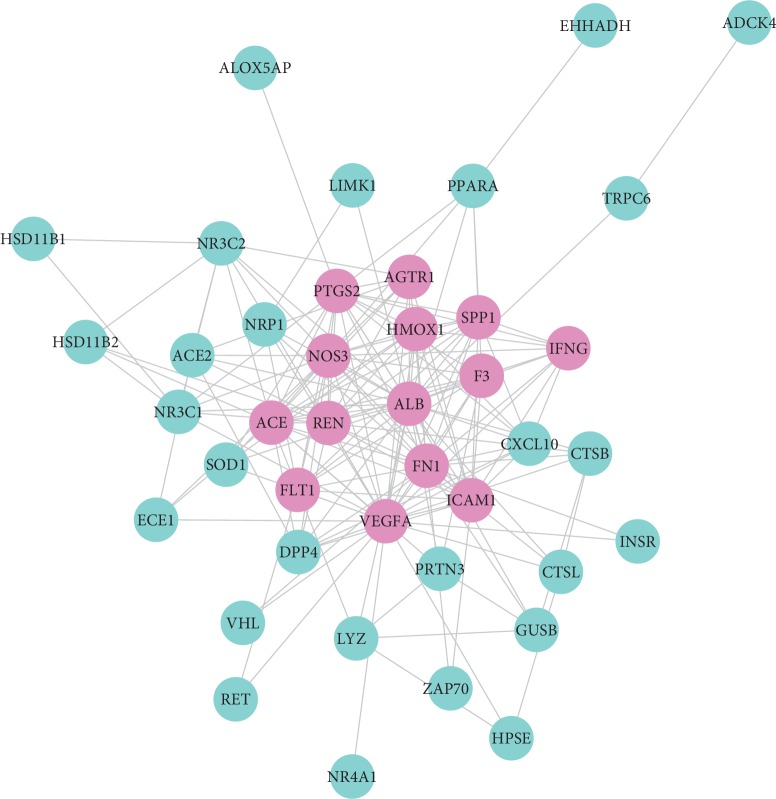
The “Candidate Targets-Proteins” network; the red nodes represent candidate targets related proteins with degree greater than or equal to 10, and the blue nodes represent the degree of proteins less than 10.

From the String database of “multiple proteins” interaction of targets, we gained 172 pairs of protein interactions of candidate targets (Supplementary [Supplementary-material supplementary-material-1]). Then, a network of “candidate targets-proteins” was constructed by Cytoscape 3.7.1 software (shown in Figure 5), and specific analytical parameters through NetworkAnalyzer plugin were in the supplemental material (Supplementary [Supplementary-material supplementary-material-1]). There were 41 nodes and 172 edges in this network, in which 41 nodes represented 41 central targets.

### 3.6. Enrichment Analysis and Molecular Docking

#### 3.6.1. Enrichment Results

**Figure 6:**
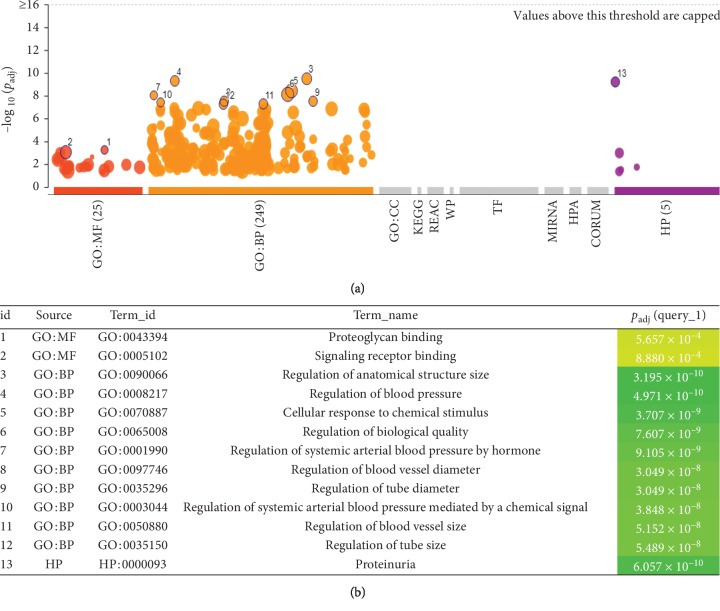
The results of the enrichment result; red points: id 1 and id 2 are the top 2 of GO molecular function; orange points: id 3 to id 12, the top 10 of GO biological process and purple points: id 13, HP results of the candidate targets.

The HP enrichment analysis indicated that the most relevant human disease of the central targets was proteinuria (Figure 6, id 13).

The results of the GO term-molecules function showed that all the central targets were associated with proteoglycan binding and signal receptor binding (Figure 6, id 1 and id 2). The GO term-biological process showed that these targets were highly related to systemic arterial blood pressure by RAS system and so forth (Figure 6, id 3 to id 12).

**Figure 7:**
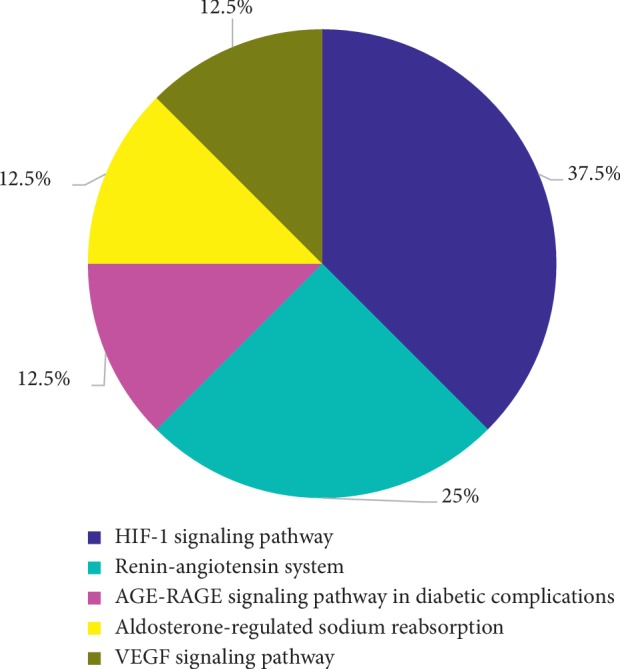
KEGG pathway enrichment results of the candidate targets. Blue, light blue, pink, yellow, and brown represent the HIF-1 signaling pathway, the renin-angiotensin system, the AGE-RAGE signaling pathway, aldosterone-mediated sodium reabsorption, and VEGF signaling, respectively.

The KEGG pathway enrichment results showed that these central targets were mainly concentrated in the hypoxia-inducible factor-1 (HIF-1) signaling pathway (37.5%), renin-angiotensin system (RAS) signaling pathway (25%), advanced glycation end products-receptor of advanced glycation end products (AGE-RAGE) signaling pathway (12.5%), aldosterone-regulated sodium reabsorption (12.5%), and vascular endothelial growth factor (VEGF) signaling pathway (12.5%) (see Figure 7).

#### 3.6.2. Molecular Docking

**Figure 8:**
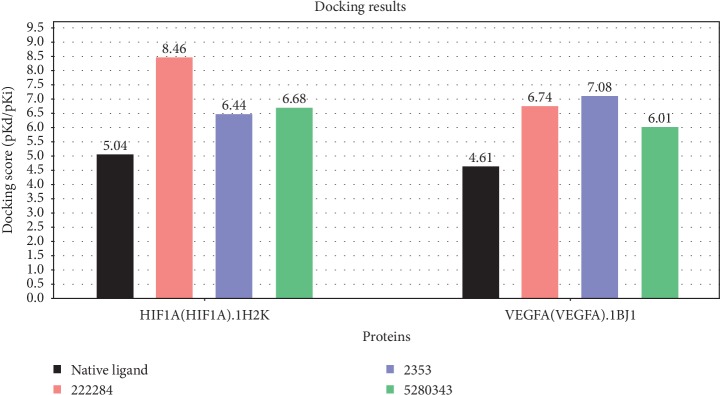
Results of the 3 major active compounds docking with 2 factors in SystemsDock. Black, pink, blue, and green column charts represent the score of native ligands, beta-sitosterol (222284), berberine (2353), and quercetin (5280343), respectively.

**Figure 9:**
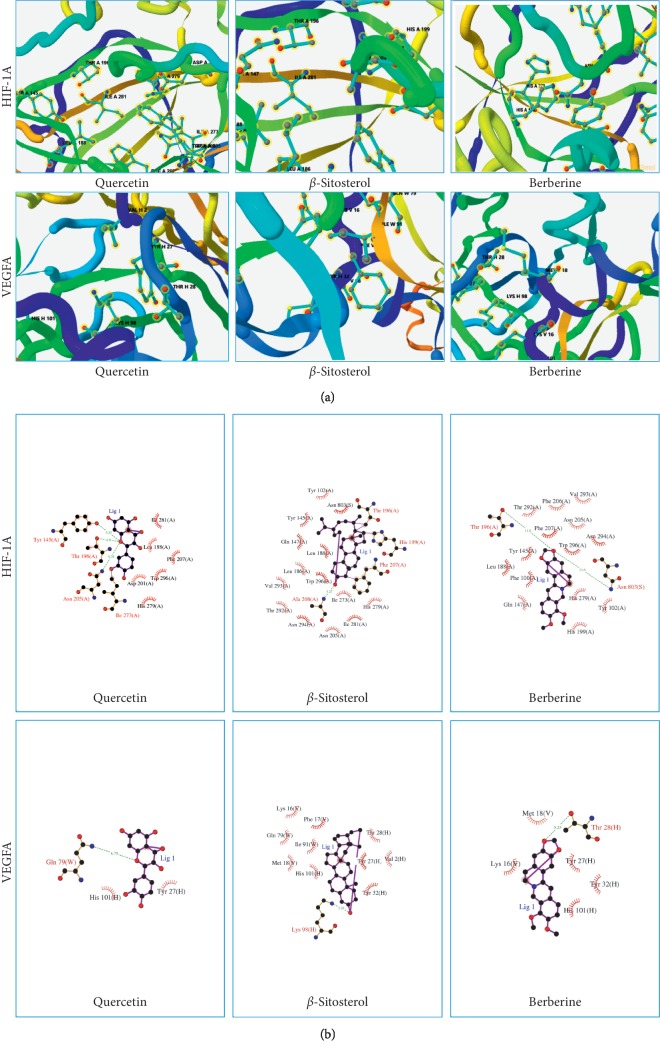
Results of the 3D and 2D docking models of 3 major active compounds docking with 2 factors in SystemsDock. (a) 3D Docking model, quercetin, *β*-sitosterol, and berberine are shown as stick with blue, and HIF-1A and VEGFA are represented as strips. (b) 2D docking model, 3 major active compounds are shown as stick with purple, and residues of 2 factors are represented as pink curves.

Molecular docking of bate-sitosterol, quercetin, and berberine with HIF-1A and VEGFA, respectively, all docking scores were not only greater than local ligand scores, but also greater than 5.52 (shown in Figure 8). The results showed that three major active ingredients could be well binding with those two factors, respectively. The 3D binding mode of 3 major active ingredients in the active site of HIF-1A and VEGFA, respectively, is represented in Figure 9(a), while schematic 2D representation is shown in Figure 9(b).

### 3.7. TLC Validation Results

**Figure 10:**
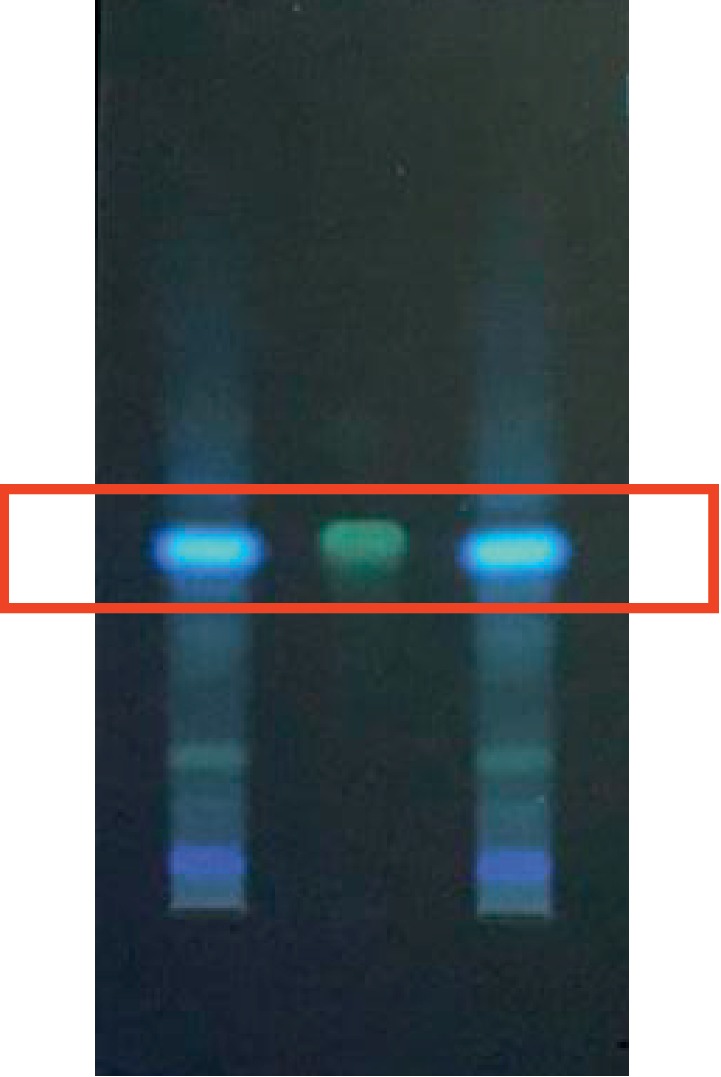
Results of TLC validation of quercetin in SBT. Middle line is the fluorescent point of the quercetin standard sample, and the SBT sample is on both sides.

In the TLC chromatogram, there are fluorescent spots in the chromatogram of the SBT sample corresponding with quercetin standard sample both in position and color (shown in Figure 10).

## 4. Discussion

Network pharmacology is a mechanism research method for TCM, based on the characteristics of the comprehensive regulation of “multi-ingredient, multitarget, and multipathway” [59–62]. In this study, synergistic pharmacological mechanisms of SBT against proteinuria were identified through 3 major active ingredients (beta-sitosterol, quercetin, and berberine), 48 candidate targets including 3 key targets and 3 pathways including HIF-1 signaling pathway, VEGF signaling pathway, RAS signaling pathway, and AGE-RAGE signaling pathway. In particular, those candidate targets were highly correlated with proteinuria in our results of HP enrichment, and those 3 major active ingredients were capable of binding to HIF-1A and VEGFA protein. Together with one of the major active ingredients, quercetin in SBT was discriminated by TLC, and all the results confirmed the reliability of our prediction.

### 4.1. Beta-Sitosterol, Quercetin, and Berberine as the Major Active Ingredients of SBT

In our study, beta-sitosterol contained in 9 herbs (HMM and PRA), quercetin contained in HMM and PPL, and berberine contained in ABR acted on 15, 11, and 11 candidate targets against proteinuria, respectively. In addition, 92.1% of 118 active ingredients acted on at least two candidate targets (Figure 4). More importantly, the three major active ingredients had tighter combinations than the local ligand with HIF-1A and VEGFA in our docking results, which demonstrates that SBT worked against proteinuria through a multi-ingredient synergistic way.

Previous studies have demonstrated that beta-sitosterol has been widely used in the treatment of proteinuria resulting from diabetes and kidney disease [63–65]. Berberine has also been reported to treat proteinuria and kidney damage caused by diabetes and hypertension [66–68], which provided evidence for our results.

It is worth mentioning that quercetin in SBT was detected in our TLC results, and studies confirmed it was able to treat proteinuria as well as improve renal function through regulating HIF-1A and VEGFA factors in rat models [69–71], which is in line with our docking results. Interestingly, other active compounds such as Astragaloside IV (contained in HMM) and paeoniflorin (contained in PRA) with a concentration of 3.20–64.00 *μ*g·mL^−1^ were detected in SBT in our previously quality standard study [72]. Studies have experimentally proved that Astragaloside IV was able to reduce proteinuria in diabetic nephropathy mice [73] and paeoniflorin could improve proteinuria in chronic renal failure rats [25]. All of the above supports our result that SBT worked against proteinuria in a multi-ingredient way.

### 4.2. PTGS2, ESR1, and NOS2 as the Key Targets of SBT against Proteinuria

In our study, PTGS2, ESR1, and NOS2 were overlapped by 74, 59, and 37 active ingredients, respectively. Moreover, 68.75 % of 48 candidate targets of SBT can be overlapped by at least two active ingredients in SBT (Figure 4), which demonstrated the active ingredient in SBT worked against proteinuria through a multitarget synergistic way.

It is reported that PTGS2 (also known as COX-2) was involved in podocyte injury and various renal pathological processes such as renal interstitial fibrosis, showing a potential therapeutic target against lupus nephritis-induced nephropathy [74, 75]. Coincidentally, several studies have proved that PTGS2 was inhibited by beta-sitosterol, quercetin, and berberine inhibiting the expression of PTGS2 in mouse colitis, the small intestine mucosa of rats, and human endometrial cells, respectively [76–78]. ESR1 is a potential therapy target for proteinuria caused by IgA nephropathy through the proliferation of glomerular mesangial cells [79]. Related research showed that quercetin regulated the activity of ESR1 in HepG2 (hepatocytes) and Caco-2 (intestinal) [80].

NOS2 could produce NO which simultaneously stimulates the increased expression of COX-2 and transforming growth factor-*β*1, causing damage in renal function, vascular and structural [81]. Related scholars have confirmed that NOS2 can be inhibited by beta-sitosterol, quercetin, and berberine in insulin rats, mouse hepatocyte, and mouse 264.7 macrophages, respectively [82–85]. Other candidate targets can also play a curative role in SBT against proteinuria, such as HSD11B1 [86] and PPAPA [87].

All these studies supported our results that SBT worked against proteinuria in a multitarget synergistic way. Whether three active ingredients act against proteinuria through regulating PTGS2, ESR1, and NOS2, it needs further study.

### 4.3. HIF-1, RAS, VEGF, and AGE-RAGE Signaling Pathway Playing an Important Role in SBT against Proteinuria

The HIF-1 signaling pathway and RAS signaling pathway were identified as mechanisms of SBT against proteinuria from ClueGO results. Studies have reported that high expression of HIF-1A in renal tubular epithelial cells can aggravate glomerular hypertrophy and extracellular matrix accumulation, increasing urinary protein excretion [88]. More importantly, HIF-1 signaling mediated pathways, such as the mitogen-activated protein kinase (MAPK) signaling pathway, and the phosphatidylinositol-3-kinase protein kinase B (PI3K-Akt) signaling pathway [89] both directly damaged renal function through contributing to renal fibrosis or proteinuria-induced renal epithelial-mesenchymal transition [90–92]. Fortunately, berberine was reported to activate HIF-1A to adapt to the stressful conditions and protected tubular epithelial cells from apoptosis in diabetic nephropathy rats [93]. However, it needs further study of whether berberine worked against proteinuria through activating HIF-1A.

In addition, many studies [94–97] have confirmed that the RAS system is closely related to proteinuria caused by various diseases, such as diabetic nephropathy and CKD. CKD is associated with hypoxia which can activate HIF-1 [98] and induce profibrogenic changes in proximal tubular epithelial cells and interstitial fibroblasts, leading to further aggravation of proteinuria [99]. Additionally, it has been demonstrated that proteinuria can activate the local RAS system in the kidneys, which can then cause vasoconstriction and subsequent hypoxia [100]. To sum up, this cross talk between proteinuria and hypoxia can cause a vicious circle of kidney destruction. Therefore, it can be speculated that the inhibition of the RAS system and HIF-1 activation may be the potential treatment for proteinuria. Fortunately, it has been reported that quercetin inhibits the RAS system and inhibits the expression of growth-transforming factor-*β* which promotes glomerular sclerosis and renal tubular interstitial fibrosis in progressive nephropathy [101]. However, there is still a lack of studies on whether quercetin works against proteinuria through inhibiting the RAS system.

Inhibition of RAS can also regulate the high glucose-induced VEGF signaling pathway [102]. VEGF-A, the most specific and prominent angiogenic factor of VEGF family, has been reported to be involved in the process of angiogenesis, migration, and proliferation of endothelial cells [8, 88, 103, 104]. In addition, autocrine secretion of VEGF was caused by the interactions of AGEs and their receptor RAGE, which were new risk factors in the pathogenesis of end-stage renal disease [105, 106]. In this background, beta-sitosterol was reported to inhibit the expression of VEGF in kidney cancer rats, protecting functions in renal tissues [107]. Quercetin can also inhibit RAGE and reduce cardiovascular damage in diabetic nephropathy [108]. Therefore, it can be speculated that inhibition of VEGF and AGE-RAGE signaling pathways may be potential treatments for proteinuria.

## 5. Conclusion

In conclusion, the “multi-ingredient, multitarget, and multipathway” of SBT against proteinuria were effectively elucidated by the network pharmacology approach. Beta-sitosterol is one of the major active ingredients used to treat proteinuria caused by IgA nephropathy, by targeting ERS1 through its anti-immune effects. Berberine reduces proteinuria and kidney damage caused by diabetes and hypertension through two key targets, PTGS2 and NOS2, which improve podocyte injury. Quercetin, contained in SBT through TLC verification, reduces proteinuria by regulating HIF-1 and VEGF in nephrons. Additionally, blocking RAS, a gold standard for treating proteinuria caused by CKD, can prevent HIF-1 and prevent VEGF from being activated. Blocking AGE-RAGE to prevent the autocrine signaling of VEGF can treat proteinuria caused by diabetic nephropathy. Thus, we predicted that SBT plays a therapeutic role in proteinuria caused by IgA nephropathy, CKD, and diabetic nephropathy and prevents complications such as cardiovascular diseases. All these contribute to the utility of SBT and may provide a new drug against proteinuria. However, more experimental studies are still needed to verify these findings.

## Figures and Tables

**Table 1:** The information of the top 10 overlapped targets.

Gene	Uniport ID	Description	Active ingredient category
PTGS2	P35354	Prostaglandin G/H synthase 2	106 kinds, kaempferol, quercetin, etc.
ESR1	P03372	Estrogen receptor	83 kinds, beta-sitosterol, berberine, etc.
CYP19A1	P11511	Cytochrome P450 19A1	76 kinds, berberine, palmatine, etc.
PTGS1	P23219	Prostaglandin G/H synthase 1	75 kinds, palmatine, quercetin, etc.
AR	P10275	Androgen receptor	71 kinds, coniferin, kaempferol, etc.
ACHE	P22303	Acetylcholinesterase	67 kinds, beta-sitosterol, mairin, etc.
PPARG	P37231	Peroxisome proliferator-activated receptor gamma	66 kinds, coniferin, mairin, etc.
PTPN1	P18031	Protein-tyrosine phosphatase 1B	66 kinds, daucosterol, hesperetin, etc.
NOS2	P35228	Nitric-oxide synthase, inducible	62 kinds, sitosterol, coptisine, etc.
CA2	P00918	Carbonic anhydrase II	59 kinds, albiflorin, kaempferol, etc.

**Table 2:** The information of the top 16 active ingredients with the herb number exceeding 8.

Compound	Mol ID	Targets category	Herbs
MOL000358	Beta-sitosterol	15 kinds, NR3C1, HSD11B2, PPARA, etc.	ABR, IRH, PRA, RPR, STR, FRU, DRA, LHE, TME
MOL000098	Quercetin	11 kinds, DPP4, PPARA, PTGS2, etc.	HMM, PPL
MOL001454	Berberine	11 kinds, PTGS2, ALOX5AP, ICAM1, etc.	ABR
MOL008006	Paryriogenin A	11 kinds, NR3C1, ACE, NOS2, etc.	TME
MOL002897	Epiberberine	11 kinds, PTGS2, ESR1, ZAP70, etc.	ABR
MOL000296	Hederagenin	11 kinds, ALOX5AP, LYZ, NOS2, etc.	ASE, HMM, LHE
MOL000378	7-O-Methylisomucronulatol	10 kinds, DPP4, GUSB, RET, etc.	IRH, HMM
MOL012542	*β*-Ecdysterone	10 kinds, AGTR1, NR3C2, TNF, etc.	ABR
MOL001494	Mandenol	9 kinds, PPARA, PTGS2, FLT1, etc.	CRH
MOL000519	Coniferin	8 kinds, AGTR1, ESR1, PTGS2, etc.	ASE, FRU, SWH
MOL000422	Kaempferol	8 kinds, ICAM1, HMOX1, DPP4, etc.	RPA, PPL, HMM, CSR, ABR
MOL000211	Mairin	8 kinds, ALOX5AP, CD81, NOS2, etc.	RPA, PPL, HMM
MOL002875	Methyl oleate	8 kinds, PPARA, PTGS2, HSD11B2, etc.	ASE
MOL000359	Sitosterol	8 kinds, ESR1, HSD11B1, NR3C2, etc.	SWH, RPA, RPR, CRH, FRU
MOL000449	Stigmasterol	8 kinds, PTGS2, ESR1, NOS2, etc.	IRH, RPR, ABR
MOL002157	Wallichilide	8 kinds, CTSL, ACE, CTSB, etc.	CRH

**Table 3:** The information of the top 10 key targets with the active ingredient number exceeding 14.

Gene	UniProt ID	Description	Active ingredients
PTGS2	P35354	Prostaglandin G/H synthase 2	74 kinds, beta-sitosterol, quercetin, berberine, etc.
ESR1	P03372	Estrogen receptor	59 kinds, beta-sitosterol, quercetin, berberine, etc.
NOS2	P35228	Nitric oxide synthase, inducible	37 kinds, beta-sitosterol, quercetin, berberine, etc.
NR3C1	P04150	Glucocorticoid receptor	29 kinds, formononetin, coptisine, baicalein, etc.
HSD11B1	P28845	11-Beta-hydroxysteroid dehydrogenase 1	28 kinds, stigmasterol, daucosterol, delta 7-stigmastenol, etc.
PPARA	Q07869	Peroxisome proliferator-activated receptor alpha	21 kinds, formononetin, coniferin, delta 7-stigmastenol, etc.
HSD11B2	P80365	11-Beta-hydroxysteroid dehydrogenase 2	19 kinds, hederagenin, bidentatoside, mandenol, etc.
NR3C2	P08235	Mineralocorticoid receptor	19 kinds, wallichilide, epiberberine, methyl oleate, etc.
DPP4	P27487	Dipeptidyl peptidase IV	14 kinds, baicalein, quercetin, methyl oleate, etc.
NOS3	P29474	Nitric-oxide synthase, endothelial	14 kinds, isorhamnetin, coptisine, syringin, etc.

## Data Availability

The data of our research can be acquired from the Supplementary Materials uploaded with this article.

## Supplementary Materials

Supplementary Materials Supplementary Table 1: 118 active ingredients in Shuangbai Tablets. Supplementary Table 2: 8981 targets of the active ingredients in Shuangbai Tablets. Supplementary Table 3: 110 523 genes associated with proteinuria. Supplementary Table 4: candidate targets with therapeutic proteinuria. Supplementary Table 5: 172 pairs of protein interactions of candidate targets. Supplementary Table 6: specific analytical parameters of 41 central targets.
